# A Microgel Platform Enables Site‐Specific Intestinal Delivery of Lactoferrin, Improving its Bioavailability for Targeted Alleviating Liver Injury and Colitis

**DOI:** 10.1002/advs.75697

**Published:** 2026-05-13

**Authors:** Huiling Yan, Yixuan Li, Shanan Chen, Pengcheng Du, Kaiwen Wu, Hui Zhang, Kasper Hettinga, Lina Zhang, Gergely Toldi, Fazheng Ren, Yuan Li

**Affiliations:** ^1^ Key Laboratory of Precision Nutrition and Food Quality Department of Nutrition and Health China Agricultural University Beijing China; ^2^ Research Center of Food Colloids and Delivery of Functionality College of Food Science and Nutritional Engineering China Agricultural University Beijing China; ^3^ Division of Food Quality and Design Wageningen University and Research Wageningen Netherlands; ^4^ School of Food Science and Technology Jiangnan University Wuxi Jiangsu China; ^5^ Liggins Institute University of Auckland Auckland New Zealand

**Keywords:** alcoholic liver injury, *artemisia sphaerocephala* krasch, bioavailability, lactoferrin delivery, microgels, polysaccharide, ulcerative colitis

## Abstract

Oral delivery of bioactive proteins remains a challenging area, as it demands effective gastric protection and intestinal site‐specific release. We design two intestinal site‐specific microgels via Fe^3^^+^‐COO^−^ coordination or STMP‐conjugated cross‐linking of eco‐friendly and biocompatible *Artemisia sphaerocephala* Krasch. polysaccharides (ASKP) extracted from desert sand‐fixing plant; these microgels release lactoferrin (Lf) precisely in the small intestine or colon on‐demand. Both microgels protect Lf from gastric digestion, preserve the Lf receptor (LfR)‐binding domain, and facilitate efficient LfR‐mediated endocytosis of Lf by intestinal epithelial cells. Small intestine‐targeted microgels regulate Lf metabolism, boost Lf bioavailability by ≈6‐fold, and drive marked hepatic Lf accumulation. These effects ameliorate alcoholic liver injury via the Nrf2‐mediated antioxidant pathway and CPT1A‐amplified fatty acid β‐oxidation after oral uptake. Colon‐targeted microgels alleviate ulcerative colitis by suppressing the TLR4/MyD88/NF‐κB inflammatory axis and restoring gut microbiota homeostasis. Notably, this is the first demonstration of site‐specific intestinal delivery achieved by using different cross‐linking chemistries on the same ASKP backbone, enabling distinct gut‐regional specific therapeutic actions. This work establishes food‐grade microgel platforms as promising intestinal site‐specific delivery systems for spatiotemporally controlled, on‐demand delivery of bioactive proteins.

## Graphical abstract

This work first time reports an eco‐friendly desert plant food‐based polysaccharide ASKP microgels which have intestinal site‐specific release properties which can achieve small‐intestine or colon targeting capability via different cross‐linking chemistry. ASKP microgels protect lactoferrin from gastric degradation, increase oral bioavailability approximately 6‐fold, maximizing in vivo health benefits against alcoholic liver disease and ulcerative colitis via intestinal site‐specific delivery.

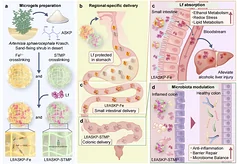

## 1 Introduction

Lactoferrin (Lf) is an iron binding glycoprotein initially isolated from milk having many health benefits such as antimicrobial, anti‐inflammatory, immunomodulatory, antioxidant and antineoplastic properties [1, 2]. The Lf from bovine milk possesses a molecular weight of around 78 kDa, while Lf from human milk is approximately 80 kDa. Lf from other species such as murine, caprine, and porcine generally fall within the range of 75–85 kDa, with variations primarily due to differences in both amino acids and glycosylation patterns [3]. Many studies have shown that the integrity and tertiary structure of Lf are highly important for its physiological bioactivity because only Lf with the proper domain, which can specifically bind to cellular receptors, can mediate associated molecular pathways [4, 5, 6, 7, 8, 9]. For example, when Lf is administered orally, it needs to bind to a specialized lactoferrin receptor (LfR) on the apical surface of intestinal epithelial cells, undergo receptor‐mediated endocytosis, and be transferred into the bloodstream for distribution to peripheral tissues [4, 5, 6]. Besides, Lf binds with receptors such as intelectin 1 (ITLN 1) which participates in intestinal iron absorption. Moreover, Lf binds with low‐density lipoprotein receptor related protein 1 (LRP1) which mediates hepatic uptake and contributes to transcytotic transport across physiological barriers [7, 8, 9]. Furthermore, Lf‐receptor engagement can further modulate the genes to tissue repairing and host regulation [1, 2, 10]. Lf is involved in numerous biochemical processes, some of which require relatively intact binding domains [4, 11, 12, 13]. Therefore, finding materials that protect the integrity of orally administered Lf and to investigate the effect of such materials on Lf absorption, metabolism, biodistribution and organ‐specific bioactivity is highly important.

To improve protease resistance, intestinal residence time and bioactivity of functional proteins (such as Lf) various delivery systems have been explored such as lipid vesicles and hydrogels [14, 15, 16, 17, 18, 19, 20, 21]. The site‐specific delivery of Lf to either the small intestine or the colon may have different physiological effects [13, 14, 15, 16, 17, 18, 19, 20]. High absorption of Lf at the small intestine may result in high amounts of Lf entering into the bloodstream and reaching other organs. Alternatively, if more Lf reaches the colon, it may modulate gut microbiota composition and activity, producing local metabolic and immunological effects [22].Thus, segmental‐specific delivery can have distinct outcomes: absorption in the small intestine elevates systemic Lf modulating immunity, whereas colon‐targeted delivery enriches luminal Lf, promoting beneficial bacteria such as *Bifidobacterium*, and suppressing NF‐κB‐mediated inflammation [23]. The site‐specific delivery can thereby lead to an improved organ specific therapeutic efficacy. Although current delivery systems can improve the stability and bioactivity of Lf to a certain extent, such as the enhanced Lf bioavailability by silk sericin hydrogels, targeted intestinal infection by alginate microparticles, and improved oral absorption and anti‐inflammatory effects via liposomal or nanoparticle formulations, intestinal delivery systems that can specifically target the small intestine or colon have not been investigated to date [14, 19, 20, 24].

**FIGURE 1:**
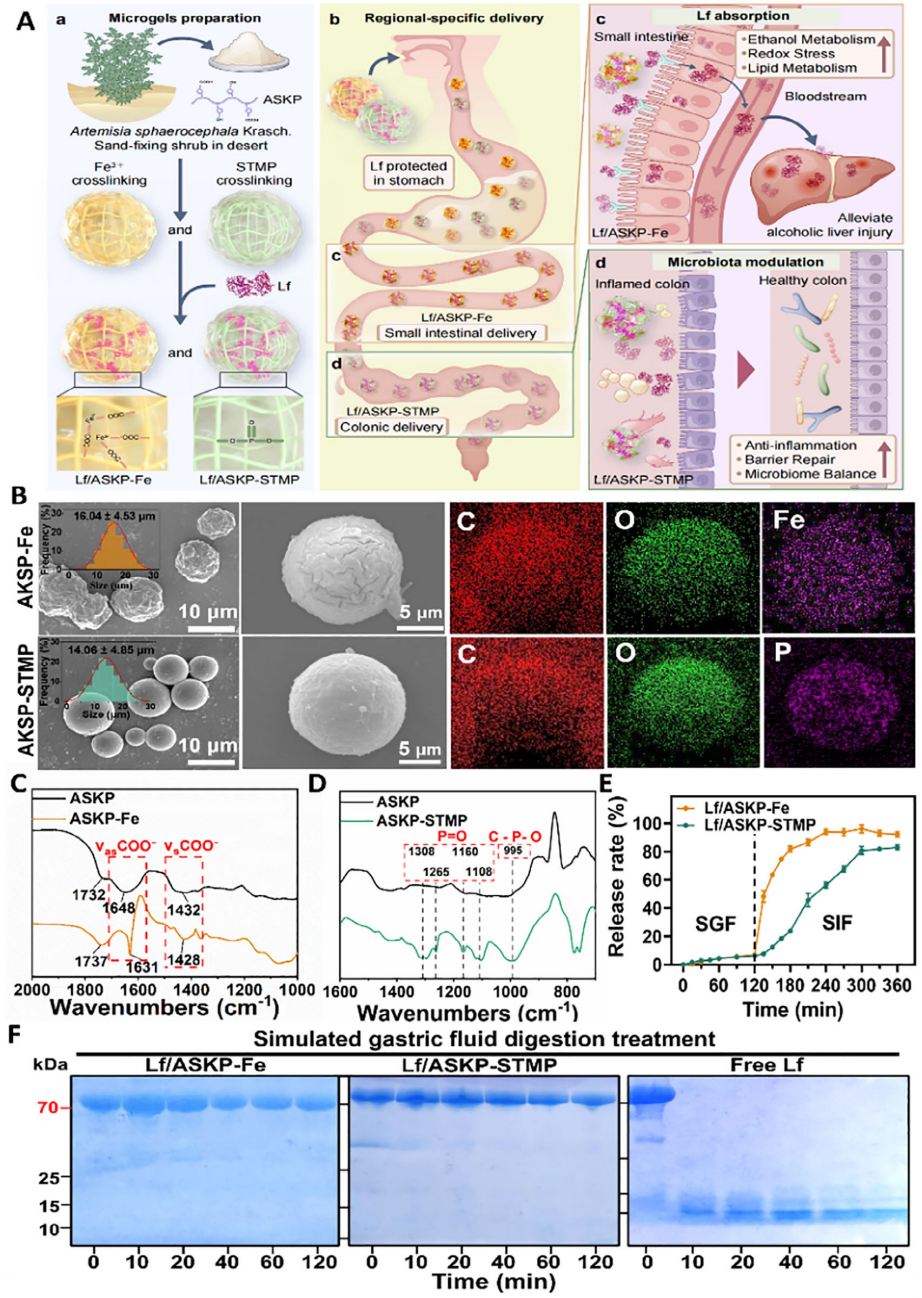
Synthesis, characterization, and gastrointestinal release profiles of ionically and covalently crosslinked ASKP microgels carrying Lf. (A) Schematic illustration of the synthesis and working mechanism of Fe^3^^+^/STMP crosslinked microgels for Lf delivery: (a) Schematic synthesis of Fe^3^^+^‐crosslinked (ionic) and STMP‐crosslinked (covalent) microgels, and Lf loading processes. (b) Protection of both microgels in the stomach and their segmental‐specific release profiles in the intestine. (c) Schematic illustration depicting the pathway of Lf/ASKP‐Fe for the release of Lf in the small intestine to its cellular uptake into the bloodstream and subsequent delivery to the liver. (d) Release of Lf/ASKP‐STMP in the colon leading to amelioration of inflammatory responses. (B) SEM image and EDS elemental mapping of ASKP‐STMP and ASKP‐Fe microgels; size distribution of ASKP‐STMP and ASKP‐Fe microgels. (C,D) FT‐IR spectra of (C) ASKP, ASKP‐Fe, and (D) ASKP‐STMP. (E) In vitro Lf release profiles in SGF and SIF. (F) SDS‐PAGE analysis of Lf released from Lf/ASKP‐STMP and Lf/ASKP‐Fe after gastric digestion for 2 h.

To address this challenge, we developed oral delivery microgels that should specifically target the small intestine and colon based on a particular type of natural polysaccharide (ASKP) (Figure 1A) extracted from the seeds of *Artemisia sphaerocephala* Krasch, a sand‐fixing plant growing in the desert of northwest China [25]. ASKP contains mannose, galactoses, rhamnose active sugar units which may work as prebiotics for modulating the gut microbiota balance. Besides, ASKP is an acidic heteropolysaccharide with abundant carboxyl and hydroxyl groups that permit diverse crosslinking chemistries [25, 26, 27, 28]. Two microgels with distinct crosslinking strategies were designed to have responsive chemical bonds: an ASKP‐Fe microgel formed through Fe^3^^+^ coordination, that should feature neutral‐pH‐responsive release in the small intestine, and a microgel covalently crosslinked with STMP for sustained release in the colon.

ASKP‐Fe microgels structurally protect Lf, efficiently delivering it via receptor‐mediated endocytosis in the small intestine, after which it accumulates in the liver, showing clear amelioration of alcoholic liver injury. The sustained release of Lf from ASKP‐STMP microgels showed to be effective in alleviation of ulcerative colitis. Together, these results demonstrate that ASKP gels can serves as a promising intestinal segmental‐specific delivery platform in terms of place, enabling on‐demand delivery of structurally intact bioactive proteins for treatment of region‐specific diseases.

## 2 Results and Discussion

### 2.1 Characterization of Ionically and Covalently Crosslinked ASKP Microgels with Intestinal Site‐Specific Controlled Release Properties

To achieve the small intestine or colon site‐specific delivery of Lf after oral uptake, two ASKP natural polysaccharides‐based microgels, responding to small intestinal or colonic conditions, were designed. The acidic heteropolysaccharide ASKP contains 5.8% uronic acids as its repeating units (Figure S1 and Table S1), providing a basis for cross‐linking by metal coordination and for electrostatic attraction to bind Lf. Specifically, an ASKP‐Fe microgel was formed via Fe^3^^+^ ionic coordination to enable neutral pH‐triggered release in the small intestine. A covalently crosslinked microgel was prepared by reacting ASKP with sodium trimetaphosphate (STMP), which is sensitive to degradation by microbial dextranases which enables sustained release in the colon (Figure 1A). The spherical microgels had an average diameter of 16.0 ± 4.5 µm for the ASKP‐Fe microgel and 14.1 ± 4.9 µm for the ASKP‐STMP microgel, respectively (Figure 1B; Figure S2). A smooth surface was observed for the ASKP‐STMP microgel, whereas a wrinkled texture was observed for the ASKP‐Fe microgel. Energy‐dispersive X‐ray spectroscopy (EDS) elemental mapping confirmed a uniform distribution of Fe in ASKP‐Fe microgel and of C, O and P in ASKP‐STMP microgel (Figure 1B). ASKP‐STMP microgel had a zeta potential of −45.98 mV while for the ASKP‐Fe microgel it was −29.72 mV, indicating the cation neutralization of the ASKP on carboxyl groups by Fe^3^^+^ (Table S2). FTIR spectra further confirmed the crosslinking mechanisms (Figure 1C,D). For ASKP‐Fe, the decreased Δ_vas‐s_ (203 vs. 216 cm^−^^1^ for ASKP) indicates a bidentate bridging coordination with Fe^3^^+^, confirming the formation of iron bridges between uronic units [29]. ASKP‐STMP showed the characteristic P═O (1308–1108 cm^−^^1^) and P─O─C (∼995 cm^−^^1^) absorption peaks [30], confirming the formation of covalent phosphate ester bonds. Lf was efficiently encapsulated into those microgels, with loading efficiencies of 258.7% (ASKP‐STMP) and 111.5% (ASKP‐Fe) (Figures S3 and S4), respectively. The 78 kDa Lf band after microgel encapsulation was confirmed by SDS‐PAGE (Figure S5). The surface hydrophobicity of Lf was progressively reduced with increasing ASKP ratio (Figure S6), likely due to the masking of Lf hydrophobic patches by absorbing ASKP polysaccharides.

Free Lf was rapidly degraded into 10–15 kDa peptide fragments within 10 min in simulated gastric fluid (SGF), whereas after 2 h in SGF Lf recovered from both microgels remained as an intact 78 kDa band on SDS‐PAGE, indicating that the microgels provided gastric protection for Lf (Figure 1F ). Consistent with the SDS‐PAGE, fluorescence microscopy (Figures S7 and S8) showed that in SGF, both microgels retained a complete structure with strong Lf (Cy5) fluorescence inside; upon transfer into SIF, Lf(Cy5)/ASKP‐Fe ruptured within 1 h and fluorescence decreased after 2 h, indicating a rapid Lf release under small intestinal conditions, whereas Lf(Cy5)/ASKP‐STMP maintained substantial high fluorescence after 2 h, confirming a sustained Lf release which may be favorable for colonic conditions. Moreover, the cumulative Lf release from both microgels was <7% after 2 h in SGF, whereas ASKP‐Fe microgel showed a rapid release after transferring into simulated intestinal fluid (SIF), while ASKP‐STMP microgel exhibited a sustained release pattern (Figure 1E,F). Our in vitro model only simulates gastric and small intestinal environments, and ASKP‐STMP's colonic degradation by microbial dextranases and subsequent Lf release were verified in vivo by small animal imaging and colonic Lf content detection. These results demonstrate the distinct release kinetics of Lf from the two microgels. The accelerated release from the ionic microgel network may be attributed to the neutral pH‐sensitive nature of Fe^3+^‐carboxylate coordination, while covalent STMP crosslinks provided a structural resistance against pH variation [29, 30]. Collectively, we successfully prepared intestinal site‐specific controlled release microgels that protect Lf against gastric digestion, with ASKP‐Fe microgel prone to Lf release in the small intestine and ASKP‐STMP in the colon, which are promising carriers for gut segmental targeted delivery of functional proteins.

### 2.2 The Cellular Uptake of Lf After Microgels Protection Under Simulated Gastric Digestion Conditions

**FIGURE 2:**
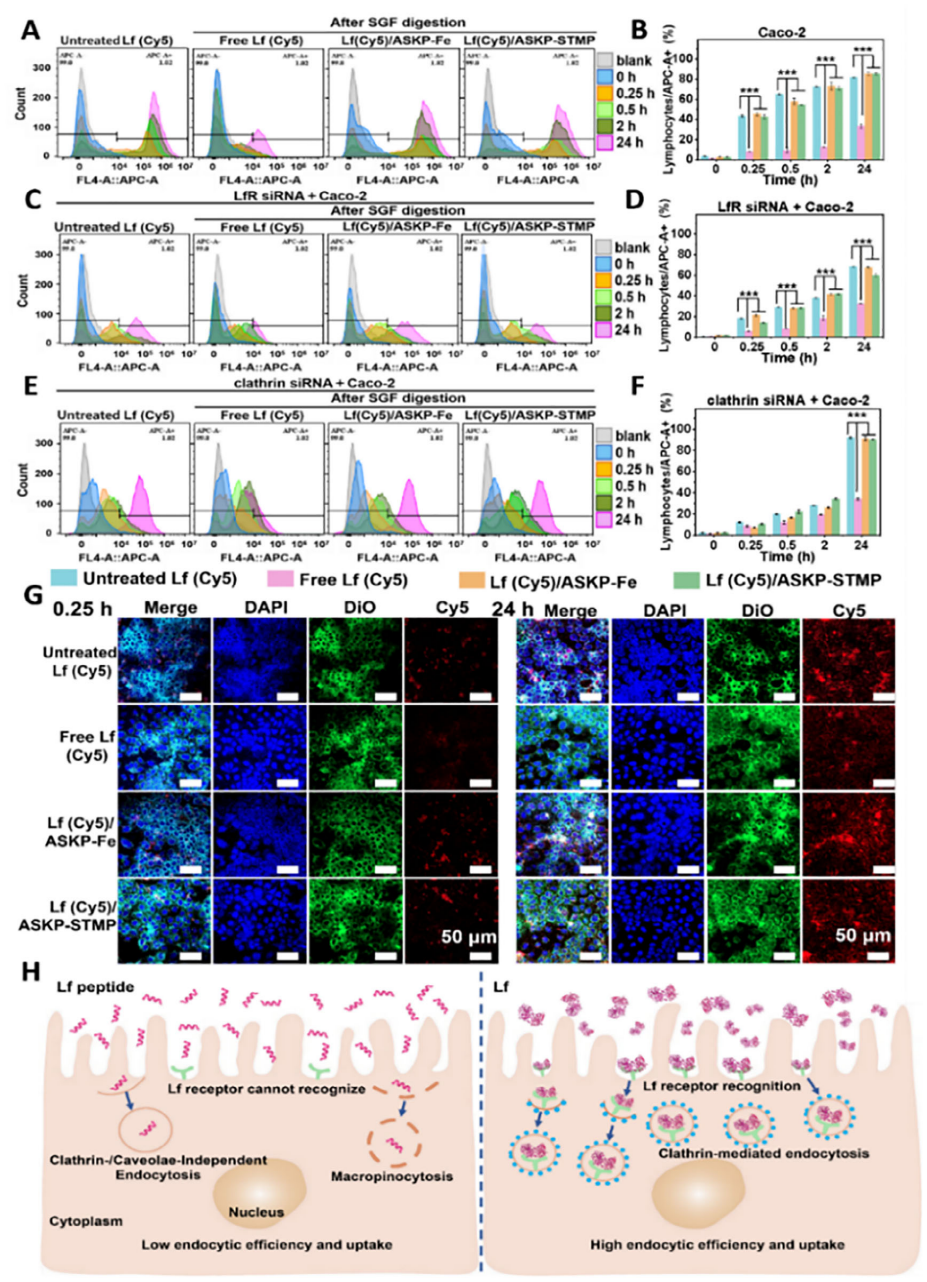
Receptor‐mediated endocytosis of Lf protected by microgels. (A) Time‐dependent fluorescence intensity of Lf vs. gastric‐digested formulations in Caco‐2 cells. Fluorescence profiles in (C) LfR‐knockdown Caco‐2 cells and (E) clathrin‐knockdown cells. Intracellular fluorescence quantification in: (B) Caco‐2, (D) siLfR, and (F) clathrin‐knockdown cells at indicated timepoints (^***^ *p* < 0.001). (G) Confocal microscopy of Lf (Cy5) internalization at 0.25 and 24 h (blue: nuclei; green: cell membrane; red: Lf (Cy5)). (H) Proposed transcellular transport mechanism of Lf via LfR‐mediated endocytosis.

To investigate the cellular uptake mechanism for free Lf and microgels loaded Lf after gastric digestion, Free Lf, ASKP‐STMP‐Lf and ASKP‐Fe‐Lf were pre‐digested for 2 h in SGF, whereas untreated Lf control was not subjected to SGF treatment. Then the ASKP‐STMP‐Lf and ASKP‐Fe‐Lf were separated and disintegrated to release loaded Lf segments. All Lf samples, including untreated Lf control, free Lf after SGF and loaded Lf after SGF digestion were labeled with Cy5 with equal protein concentration (Figure S9). All treated Lf samples were incubated with Caco‐2 cells for 0.25, 0.5, 2, and 24 h, and intracellular fluorescence was quantified by flow cytometry. Significant uptake was detected in all groups at 0.25 h (Figure 2A,B). At matched time points, intracellular fluorescence of untreated Lf, digested ASKP‐STMP‐Lf and digested ASKP‐Fe‐Lf were significantly higher than that of digested free Lf (^***^ *p* < 0.001), indicating that Caco‐2 cells had higher endocytosis rate for untreated Lf, ASKP‐STMP‐Lf, and ASKP‐Fe‐Lf, than digested free Lf (Figure 2H). CLSM imaging also showed a strong red fluorescence for untreated Lf, digested ASKP‐STMP‐Lf, and digested ASKP‐Fe‐Lf, with prominent intracellular accumulation. By contrast, free Lf after SGF produced only a weak signal restricted to the cytoplasm due to pepsin degradation (Figure 2G; Figure S10). It was reported that Lf likely entered into the epithelial cells via Lf receptor (LfR)‐mediated internalization via the recognition of the N1 domain on Lf to LfR during intestinal absorption [6, 13].

To probe the cellular uptake mechanism, the endocytosis rate of different formulations was investigated on LfR and clathrin siRNA knockdown cell lines by flow cytometry (Figure S11). Knockdown of LfR produced a time‐dependent inhibition of cellular uptake: it markedly reduced uptake of untreated Lf and Lf released from ASKP‐STMP‐Lf and ASKP‐Fe‐Lf after SGF digestion (Figure 2H). During short incubations (15–30 min), LfR knockdown caused ∼58.1% inhibition for untreated Lf, ∼67.4% for ASKP‐STMP‐Lf and ∼52.7% for ASKP‐Fe‐Lf at 15 min, with inhibition remaining ∼47.9%–55.4% at 30 min. In contrast, uptake of free Lf after SGF was only weakly affected by LfR knockdown (∼27.0% inhibition at 15 min and ∼9.4% at 30 min), consistent with digestion‐derived Lf peptides not relying on LfR‐mediated internalization (Figure 2C,D). Because clathrin‐mediated endocytosis is a common route for receptor‐dependent internalization, we performed parallel experiments in clathrin‐siRNA knockdown cells [13, 31, 32]. Clathrin knockdown produced even stronger short‐term inhibition: uptake of untreated Lf, ASKP‐STMP‐Lf and ASKP‐Fe‐Lf was reduced by ∼72.1%–84.4% at 15 min, respectively, and remained inhibited by 59.0%–70.7% at 30 min. Uptake of free Lf after SGF was hardly affected at these early times (∼5.6% inhibition at 15 min). At 2 h, uptake of untreated Lf, ASKP‐STMP‐Lf and ASKP‐Fe‐Lf remained substantially inhibited (∼42.9%–64.4%), supporting a dominant role for clathrin‐dependent uptake for these samples. At 24 h, dependence on LfR and clathrin had markedly decreased across all groups (Figure 2E,F). Together, the above results indicate that LfR and clathrin jointly mediate the cellular uptake of untreated Lf and microgels loaded Lf at an early stage, with the Lf were remained by microgels encapsulation. Gastric digestion, which likely disrupts the N1 domain of free Lf [13], weakens this dependence on receptor‐/clathrin‐mediated endocytosis thus reducing its endocytosis by Caco‐2 cells. The proposed mechanism is summarized in Figure 2H. Although hydrolyzed Lf peptides are smaller and can potentially enter cells via alternative pathways such as peptide transporters or passive diffusion, the efficiency of LfR‐ and clathrin‐dependent active endocytosis are still higher [33, 34, 35]. Overall, both microgels increased Lf endocytosis via protecting the LfR‐binding domain against digestion.

### 2.3 In Vivo Biodistribution and Bioavailability of Lf Loaded Microgels

**FIGURE 3:**
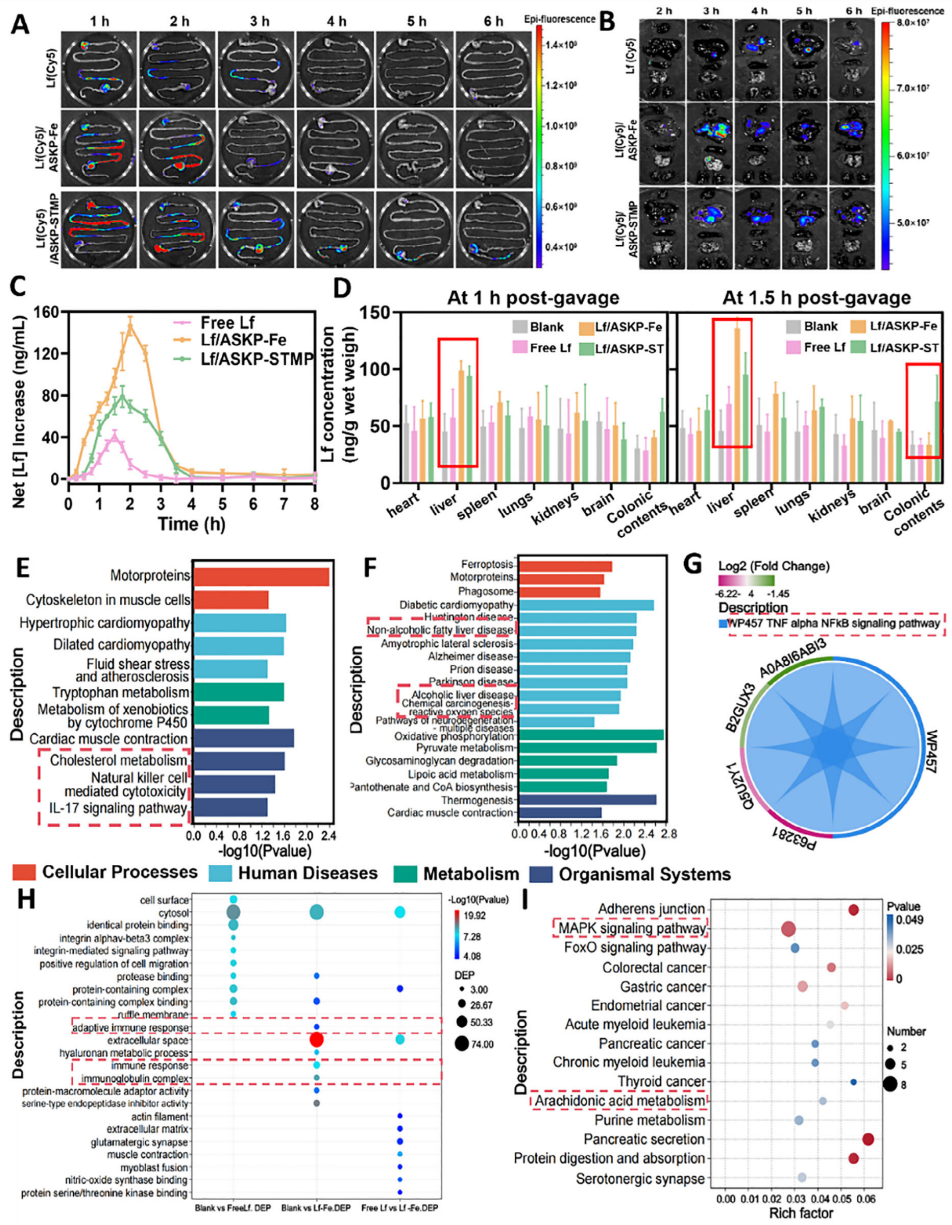
Comprehensive Analysis of Lf/Microgels: Biodistribution, Bioavailability, and Proteomic Responses in Serum and Liver. (A,B) In vivo fluorescence imaging of Lf biodistribution. (A) Gastrointestinal tract at different time points post‐administration. (B) Accumulation in visceral organs (heart, liver, spleen, lung, and kidney). (C) Concentration‐time profiles of the net increase in plasma Lf after oral administration of free Lf, Lf/ASKP‐STMP, and Lf/ASKP‐Fe microgels. (D) Quantification of Lf in organs and colonic contents by ELISA at 1 h and 1.5 h post‐oral gavage in mice. (E) KEGG pathway plot for upregulated differential proteins (Free Lf vs. Lf/ASKP‐Fe) in serum. (F) KEGG pathway plot for downregulated differential proteins (Free Lf vs. Lf/ASKP‐Fe) in serum. (G) Chord diagram of downregulated proteins from WikiPathways enrichment (Free Lf vs. Lf/ASKP‐Fe) in serum. (H) Advanced enrichment bubble plot of differential proteins for pairwise serum comparisons among Blank, Free Lf and Lf/ASKP‐Fe. (I) KEGG enrichment analysis ——downregulated pathways (Free Lf vs. Lf/ASKP‐Fe) in liver.

To track the effect of encapsulation on the biodistribution of orally ingested Lf along the gastrointestinal tract and liver, free and encapsulated Cy5‐labeled Lf (at equal Lf concentration) were gavaged to mice, and their distribution was subsequently examined by an animal fluorescence imaging system. The fluorescently labeled Lf was clearly observed after 1 h post‐gavage (Figure 3A). The fluorescent signal from free Lf(Cy5) was the weakest and nearly disappeared at 2 h probably due to the gastrointestinal digestion while the microgels loaded with Lf showed a much stronger fluorescent signal. Interestingly, Lf(Cy5)/ASKP‐Fe localized more to the ileum sites, which indicates a high absorption of Lf in the small intestine. In contrast, after 2 h Lf(Cy5)/ASKP‐STMP had reached the distal small intestine and continued toward the colon. Lf(Cy5)/ASKP‐STMP was present in after 3 h and showed a more sustained‐release profile for 6 h, releasing Lf probably during colonic fermentation. Intestinal sections (Figure S12) confirmed the in vivo results: the epithelial Cy5 signal was weaker for free Lf but stronger for Lf from ASKP‐Fe (mainly proximal small intestine) and ASKP‐STMP (extending into distal small intestine and colon), consistent with a distal, sustained release for ASKP‐STMP microgel. Given that differences in intestinal absorption efficiency may drive distinct sites targeting, logically, Lf/ASKP‐Fe yielded the strongest hepatic signal compared with Lf(Cy5)/ASKP‐STMP and free Lf (Figure 3B), indicating that the ASKP‐Fe microgel promoted Lf to be transported across the intestinal epithelium and being absorbed more in blood and subsequently being taken up by the liver.

To further study the impact of microgel on relative Lf bioavailability, pharmacokinetic studies comparing oral administration of free Lf, Lf/ASKP‐Fe and Lf/ASKP‐STMP at equal Lf concentrations were performed. Following background correction, the concentration‐time profiles depicting the net increase of exogenous bovine Lf in serum for both encapsulated and non‐encapsulated formulations are presented in Figure 3C [19, 24]. The experimental results demonstrate that Lf was detectable in the serum of all SD rat groups within the 0–8 h period after a single‐dose oral gavage administration. Compared with the free Lf, Lf encapsulated in microgels significantly enhanced the relative oral Lf bioavailability. The oral bioavailability and maximum serum Lf concentration for Lf/ASKP‐Fe and Lf/ASKP‐STMP microgels were 593% (*C_max_* = 146.3 ng/mL) and 339% (*C_max_* = 78.8 ng/mL), respectively (Table S3). These findings indicate that microgel encapsulation effectively protects Lf from degradation by gastric pepsin, thereby improving its oral bioavailability. After oral administration of equal relative amounts of Lf, mouse organs were homogenized and Lf concentration was quantified using a double‐antibody sandwich ELISA (Figure 3D; Figure S13). The results were consistent with in vivo imaging (Figure 3B): the liver was the primary site of accumulation, with Lf/ASKP‐Fe showing significantly higher levels than other groups at 1–2 h, while Lf/ASKP‐STMP exhibited the highest levels in colonic contents at 1.5–2.5 h. No significant increases were observed in other tissues (heart, spleen, lung, kidney, brain). To avoid an allergic reaction, the treated Lf concentration was controlled to be within a low range. Lf has been reported to exhibit low allergenicity, as no allergic responses were observed in rats following repeated oral administration of Lf at doses as high as 1000 mg/kg body weight for 28 days or 2000 mg/kg body weight per day for 13 weeks [36, 37]. The serum Immunoglobulin E (IgE) levels remained in the normal range (with no significant elevation compared with the control group) during 16 days of oral Lf formulations treatment once daily by oral gavage (200 µL per mouse, 300 mg/kg Lf equivalent) (Figure S14), suggesting that oral Lf treatments from our dosage did not induce systemic immune sensitization or pose an allergic risk. As illustrated in Figure 1A, microgels are designed to have specific modulatory properties that govern the release kinetics of Lf. Rapid release in the small intestine (Lf/ASKP‐Fe) results in higher systemic absorption and hepatic enrichment, whereas sustained release (Lf/ASKP‐STMP) allows a large fraction of Lf to reach the colon. These findings demonstrate that regulating release profiles through microgel design protects Lf from gastric degradation and provides a site‐specific release function which can modulate Lf absorption and metabolism.

### 2.4 Lf Metabolism and Their Host Proteome Responses Modulated by Microgels

The previous results showed that ferric ions cross‐linked ASKP microgels released Lf earlier in the small intestine, whereas STMP cross‐linked ASKP microgels provided a sustained release in the colon. The microgels thus modulate Lf release patterns which may further affect Lf metabolism and host proteome responses. Based on pharmacokinetic data (Figure 3C), Lf/ASKP‐Fe achieved the highest bioavailability, which is 5.9‐fold higher than Free Lf. To further characterize Lf after oral administration, the time of peak Lf concentration in blood (T_max_) was analyzed by proteomic techniques using LC‐mass spectrometry (MS). These results indicate the presence of Lf in serum (Table S4 and Figure S15), confirming the absorption of orally administered Lf from microgels into the systemic circulation. Lf is a ubiquitous endogenous protein in mammals. Accordingly, endogenous Lf was also detected in the blank group of this study. Furthermore, proteomics MS identified an Lf‐derived peptide fragment with a molecular weight of approximately 38.9 kDa in serum (Table S4 and Figure S16). It is postulated that a portion of the Lf underwent metabolism, either during oral absorption or after entering the systemic circulation. Due to instrumental detection limits, smaller Lf peptide fragments cannot be detected due to the measurement limit. Consistent with these findings, proteomics analysis revealed a higher enrichment of Lf in liver tissue from the Lf/ASKP‐Fe group compared to the free Lf group (Figures S17 and S18). Quantitative analysis, corrected for the influence of endogenous Lf, indicates that the relative level of Lf in the liver of the Lf/ASKP‐Fe group was 3.54 times higher than that of the free Lf group.

In spite of analyzing the serum Lf profile, proteins were performed to examine the host protein responses after various treatments. In serum, 3503 proteins were reliably quantified. Differential expression analysis (*p* < 0.05, fold change >1.2) revealed hundreds of proteins altered by Lf/ASKP‐Fe compared with Free Lf (Figure S19). Among these, Lyst (involved in cytotoxic granule trafficking) [38] and Dynamin‐2 (endocytosis) [39] were upregulated, consistent with enhanced receptor‐mediated Lf uptake. KEGG pathway enrichment analysis provided functional context: Lf/ASKP‐Fe increased pathways related to lipid metabolism, innate immunity (NK cell cytotoxicity), and IL‐17 signaling, while reducing pathways associated with fatty liver disease, alcoholic liver injury, and oxidative stress (Figure 3E,F). Importantly, suppression of the TNFα‐NFκB axis by Lf/ASKP‐Fe suggests an anti‐inflammatory effect (Figure 3G). Advanced enrichment analysis (Figure 3H) highlighted terms such as “adaptive immune response” and “immunoglobulin complex” specifically in Lf/ASKP‐Fe, but not in Free Lf. Liver proteomics quantified 7,538 proteins with high‐quality data. Differential analysis identified 378, 379, and 313 DEPs for Blank vs Free Lf, Blank vs Lf/ASKP‐Fe, and Free Lf vs Lf/ASKP‐Fe, respectively. KEGG analysis (Figure 3I) showed that, Lf/ASKP‐Fe downregulated multiple cancer‐ and inflammation‐related pathways (e.g., MAPK signaling, arachidonic acid metabolism) while free Lf did not had such an effect, consistent with the reduced stellate‐cell activation and inflammatory mediator production [40, 41]. In summary, Lf/ASKP‐Fe markedly increased systemic Lf exposure, facilitated receptor‐mediated endocytosis, and altered serum and hepatic metabolism and host proteomes response, leading to the enrichment of protective immune pathways and suppression of liver disease‐related pathways.

### 2.5 The Alleviation Effect of Lf Loaded Small Intestinal Targeted ASKP Microgels on Alcohol‐Induced Liver Injury

**FIGURE 4:**
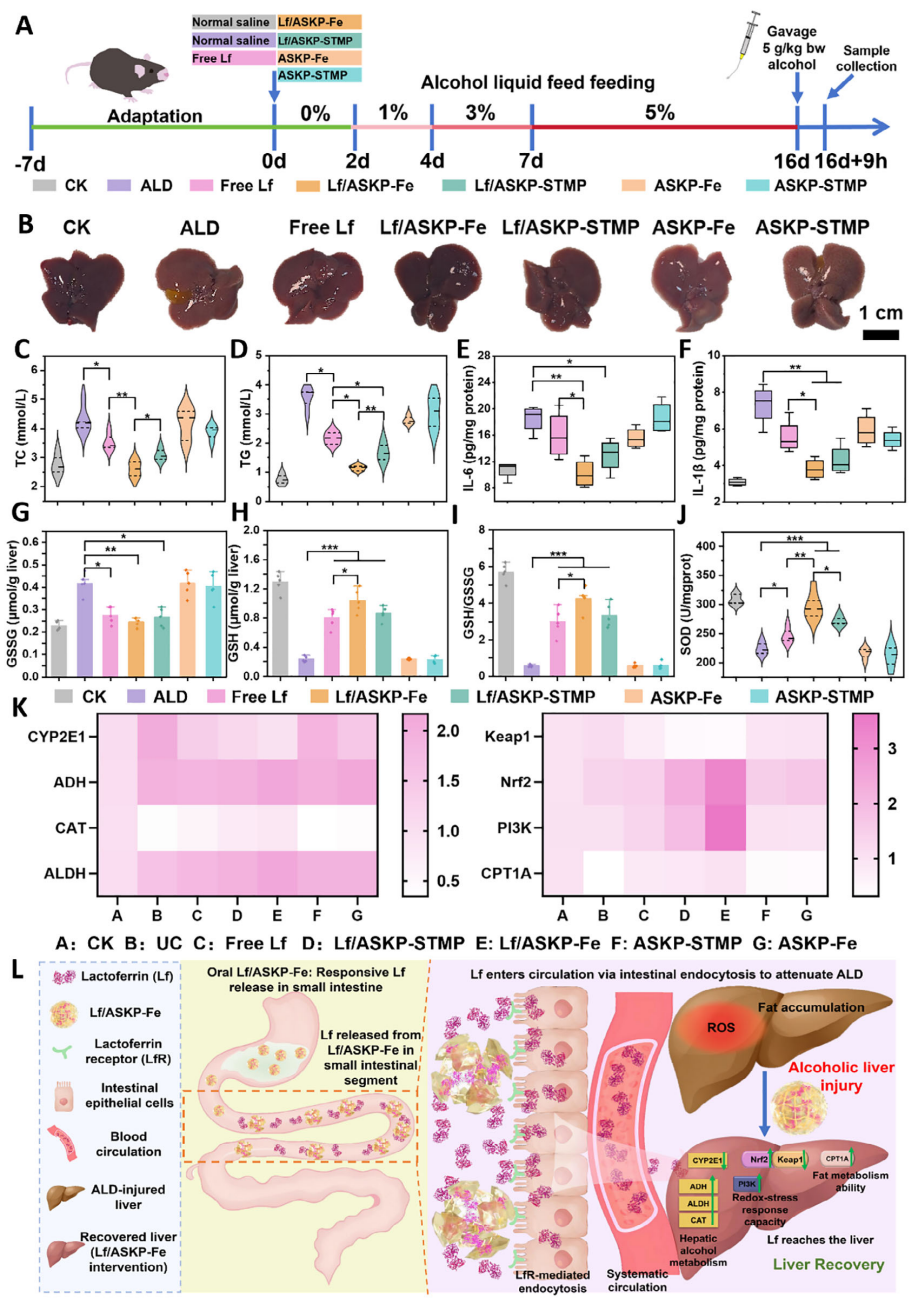
The alleviating effect of Lf loaded microgels on alcohol‐induced liver injury through antioxidant activation and metabolic reprogramming. (A) Experimental timeline of ethanol‐induced liver injury intervention. (B) Photographs of the livers of mice in each group. (C,D) Serum levels of total cholesterol (TC) and triglycerides (TG). (E‐F) Measurement of IL‐6 and IL‐1β indicators of inflammation in liver. (G–I) Measurement of oxidized glutathione (GSSG), reduced glutathione (GSH), and reduced glutathione (GSH)/oxidized glutathione (GSSG) ratio in liver. (J) Liver SOD activity. (K) qRT‐PCR of alcohol metabolism (CYP2E1, ADH, CAT, ALDH), antioxidant (Keap1, Nrf2, PI3K) genes, and lipid regulators (CPT1A). (L) Schematic diagram of the mechanism by which Lf may alleviate alcoholic liver injury. (^*^ *p* < 0.05, ^**^ *p* < 0.01, ^***^ *p* < 0.001).

The above results indicate that Lf/ASKP‐Fe and Lf/ASKP‐STMP achieved an intestinal site‐specific delivery of Lf to small intestine or colon, respectively. Lf/ASKP‐Fe released more Lf in the small intestine, which may increase their liver accumulation via intestinal absorption and blood‐liver circulation (Figure 3D). Furthermore, an alcohol‐induced liver injury (ALD) model was established. After acclimation to the ethanol diet, mice were maintained on a 5% (v/v) ethanol liquid diet. During the 16‐day experiment, animals received a once‐daily oral administration of the assigned samples (200 µL per mouse, equivalent to 300 mg Lf/kg body weight). Nine hours after a final ethanol challenge (5 g/kg body weight), mice were euthanized to evaluate treatment effects (Figure 4A) [42]. The body weight decreased in all groups during alcohol treatments (Figure S20). Compared with the ALD group, the body weight loss was significantly inhibited in Lf/microgels groups (^**^ *p* < 0.01), and Lf/ASKP‐Fe group showed the lowest weight decrease. Moreover, the liver coefficient (liver weight/body weight ratio) was significantly increased in the ALD group (^**^ *p* < 0.01, Figure S21). Surprisingly, Lf/ASKP‐STMP or Lf/ASKP‐Fe markedly reduced the liver coefficient similar as normal CK level, indicating an alleviation effect of alcohol‐induced liver enlargement. Macroscopic observation showed that livers in the ALD group were abnormally pale, possibly due to lipid accumulation, whereas liver color returned to normal after Lf/ASKP‐Fe or Lf/ASKP‐STMP treatments (Figure 4B), suggesting a negligible lipid accumulation.

Serum biochemistry confirmed severe hepatic injury (^***^ *p* < 0.001) in the ALD group, with a marked increase of alanine aminotransferase (ALT) and aspartate aminotransferase (AST), which are key indicators of liver function [43]. All Lf formulations showed reduced ALT and AST levels indicating the hepatoprotective effect of Lf. Lf/ASKP‐Fe treatment reduced ALT and AST levels the most, nearly recovering to control level (Figure S22), indicating a more pronounced hepatoprotective effect of Lf/ASKP‐Fe. H&E staining showed that Lf treatment markedly improved liver histology, and Lf/ASKP‐Fe producing superior healing effects than Free Lf and Lf/ASKP‐STMP (Figure S23). Oil Red O staining showed virtually no lipid droplets in CK group, whereas abundant lipid droplets were observed in ALD group. The lipid droplet number and size were markedly reduced in all treatment groups, and the Lf/ASKP‐Fe group almost reduced the lipid accumulation to control group. Alcoholic liver injury is characterized by a profound disruption of hepatic lipid metabolism, a hallmark of the disease that drives steatosis and disease progression [44]. The serum lipids profile further indicates the disruption of lipid metabolism in the ALD group (increased TG, TC, LDL‐C and decreased HDL‐C; Figure 4C,D and Figure S24). Free Lf and Lf/microgels treatments all reduced those abnormalities, with the largest recovery being observed in the Lf/ASKP‐Fe group, suggesting an enhanced efficacy in modulating alcohol‐induced hepatic lipid metabolic disorder.

Inflammatory cytokines were all increased in the ALD group. Free Lf and Lf/microgels significantly suppressed these proinflammatory mediators. The anti‐inflammatory cytokine IL‐10 was significantly upregulated in all treatment groups, and the Lf/ASKP‐Fe group exhibited the most prominent modulation indicating restoration of the inflammatory balance (Figure 4E,F; Figure S25). Besides, the oxidative stress‐related parameters revealed that T‐GSH and SOD activity in liver were significantly decreased, i.e., GSSG increased, and GSH and GSH/GSSG ratio markedly were reduced in the ALD group (Figure 4G–J; Figure S26), indicating severe oxidative stress. Free Lf and Lf/microgels significantly restored T‐GSH and SOD activity, and Lf/ASKP‐Fe showed a significant higher improvement in GSSG, GSH and the GSH/GSSG ratio (^*^ *p* < 0.05), indicating an enhanced mitigation of oxidative damage. Taken together, Lf/ASKP‐Fe, designed for small intestinal release, leads to a higher systemic bioavailability (Figure 3B) and enhanced transportation to the liver. Corresponding serum and liver proteomic analyses (Figure 3E–I) revealed that increased Lf exposure influenced the key metabolic and signaling pathways, including upregulation of lipid metabolism and innate immune pathways, as well as downregulation of inflammation‐ and oxidative stress‐related pathways, thus indicating a superior anti‐inflammatory, antioxidant, and lipid‐modulating effects of Lf/microgels against alcoholic liver injury.

To further investigate the mechanisms by which Lf/ASKP‐Fe ameliorates alcoholic liver injury, we examined key genes and proteins involved in alcohol metabolism, antioxidant response, and lipid metabolism [45, 46]. As schematically illustrated in Figure S27, ethanol is metabolized mainly through three pathways: alcohol dehydrogenase (ADH), the microsomal ethanol‐oxidizing system (primarily CYP2E1), and catalase (CAT) [45, 46]. In the heat map, deeper pink denotes a higher mRNA expression while colors approaching white denote lower expression (Figure 4K). CYP2E1, closely associated with ROS generation, was markedly increased in the ALD group compared with CK (approximately 2‐fold, ^**^ *p* < 0.01). Consistently, all treatment groups showed significant downregulation of CYP2E1 (^**^ *p* < 0.01), and Lf/ASKP‐Fe exhibited the most pronounced inhibitory effect. Meanwhile, ALDH mRNA was elevated after ethanol exposure, and Lf/ASKP‐Fe increased ALDH expression most significantly (^**^ *p* < 0.01), suggesting an enhanced acetaldehyde clearance.

During ethanol metabolism, the Keap1/Nrf2 antioxidant signaling axis serves as a central transcription factor for endogenous antioxidant defense, inducing cytoprotective genes such as NQO1 and HO‐1 to protect hepatocytes from oxidative stress (Figure S28) [47, 48]. Lf/ASKP‐Fe promoted Nrf2 nuclear translocation and significantly reduced Keap1 levels, thereby activating downstream antioxidant genes and attenuating ROS generation (Figure 4K; Figure S28). Concurrently, the PI3K‐Akt pathway was enhanced, as evidenced by increased phosphorylation of PI3K and Akt (^**^ *p* < 0.01), which complements the antioxidant response by upregulating protective proteins such as SOD and preserving mitochondrial integrity (Figure 4J) [49, 50]. The suggests that microgels loaded with Lf can alleviate the oxidative stress caused by the over consumption of alcohol via delivering more Lf to the liver.

Previous studies reported that Lf upregulates hepatic carnitine palmitoyl transferase 1A (CPT1A), thereby promoting long‐chain fatty acid transport into mitochondria and enhancing β‐oxidation and energy production [51]. According to literature, CPT1A expression was suppressed after alcohol exposure, and Lf can upregulate CPT1A [51]. The results showed that Lf/ASKP‐Fe upregulated CPT1A expression more than Free Lf (^**^ *p* < 0.01, Figure 4K), suggesting that hepatic delivery of Lf by ASKP‐Fe can reduce the lipid accumulation via enhanced fatty acid β‐oxidation by the CPT1A pathway. In summary (Figure 4L), small intestinal release of Lf from Lf/ASKP‐Fe enhances hepatic ethanol metabolism, alleviates oxidative stress via Nrf2 and PI3K‐Akt pathways, and promotes lipid clearance through CPT1A, collectively contributing to its potent protective effect against alcoholic liver injury.

### 2.6 The Alleviation Effect of Lf Loaded Colonic Targeted ASKP Microgels on Ulcerative Colitis

**FIGURE 5:**
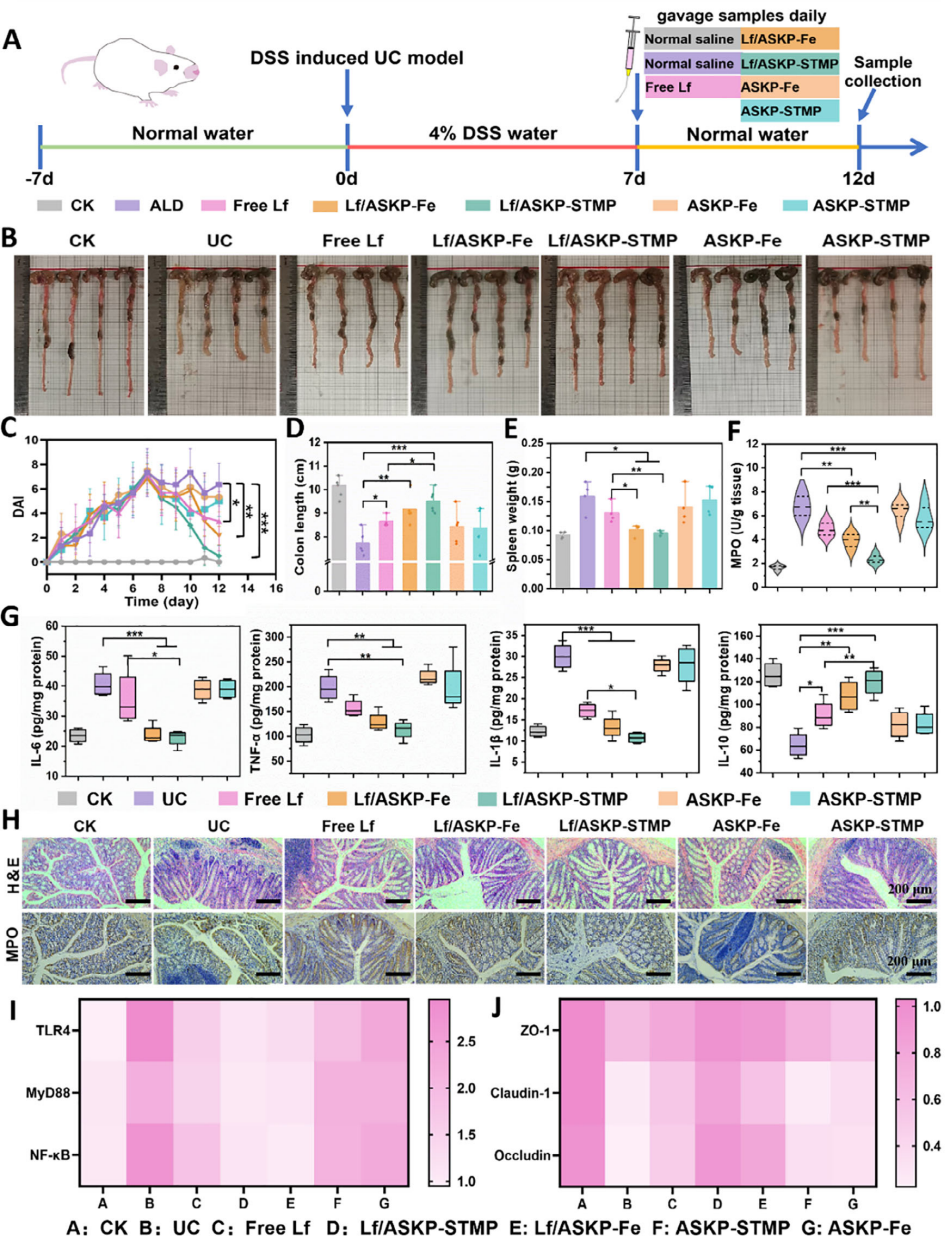
Intervention of microgels loaded with Lf on ulcerative colitis and restoring the intestinal barrier function. (A) Experimental timeline of DSS‐colitis intervention. (B) Macroscopic morphology and (D) length measurement of excised colons. (C) DAI scores during treatment. (E) Splenomegaly assessment. (F) Myeloperoxidase (MPO) activity in serum. (G) Measurement of IL‐6, TNF‐α, IL‐1β, and IL‐10 in serum. (H) Histopathological analysis: H&E staining (top) and MPO immunohistochemistry (bottom). (I) qRT‐PCR analysis of TLR4/MyD88/NF‐κB pathway mRNA expression. (J) Tight junction gene expression (ZO‐1, Claudin‐1, Occludin). (^*^ *p* < 0.05, ^**^ *p* < 0.01, ^***^ *p* < 0.001).

Since Lf/ASKP‐STMP released Lf mainly in the colon (Figure 3D), the health benefits of Lf were further confirmed by a dextran sulfate sodium (DSS)‐induced mouse model of ulcerative colitis (UC) [52, 53]. A systematic assessment was performed, including disease activity, colon morphology, and stool characteristics after various Lf/ASKP microgels treatments (Figure 5A). After 7 days of DSS administration to induce colitis, all groups except the healthy control (CK) exhibited gross bleeding and loose or unformed stools (Figures S29 and S30). Following 5 days of treatment on the UC mice, the disease activity was improved markedly in both Lf/ASKP‐STMP and Lf/ASKP‐Fe groups, and stool consistency approached that of the health groups. The DAI score, calculated from body‐weight change, stool consistency, and bleeding [52, 53], both microgels encapsulating Lf showed a significant reduction compared with the UC group (^*^ *p* < 0.05, ^**^ *p* < 0.01; Figure 5C), especially for the Lf/ASKP‐STMP group which had the lowest DAI score among all groups. Anatomical measurements revealed a pronounced colon shortening in the UC group (colon length = 76% of CK; Figure 5B,D). Both Lf/ASKP‐STMP and Lf/ASKP‐Fe substantially attenuated colon shortening, restoring colon length close to healthy levels, with Lf/ASKP‐STMP showing the best recovery. Splenomegaly, a hallmark of systemic inflammation [54, 55], was obvious in the UC group (significant increase in spleen weight; ^**^ *p* < 0.01, Figure 5E) while not occurring in both Lf/ASKP‐STMP and Lf/ASKP‐Fe groups. Free Lf produced a partial reduction in spleen weight but remained significantly higher than the healthy group and the two microgel groups (^*^ *p* < 0.05, ^**^ *p* < 0.01), indicating limited efficacy of free Lf. MPO, a surrogate marker of neutrophil infiltration and inflammatory intensity [52, 56], was markedly elevated in UC models (^***^ *p* < 0.001, Figure 5F). Lf/ASKP‐STMP reduced MPO more effectively than Lf/ASKP‐Fe, highlighting its superior anti‐inflammatory activity. Serum cytokine profiling showed a significant increase in proinflammatory IL‐6, IL‐1β, and TNF‐α in the UC group (^**^ *p* < 0.01, ^***^ *p* < 0.001). Treatment with Lf/ASKP‐STMP or Lf/ASKP‐Fe suppressed these cytokines toward healthy levels (*p* > 0.05) and significantly elevated the anti‐inflammatory cytokine IL‐10 (^**^ *p* < 0.01; Figure 5G). Obviously, Lf/ASKP‐STMP outperformed Lf/ASKP‐Fe in downregulating proinflammatory cytokines and increasing IL‐10 (^*^ *p* < 0.05). Histopathological analysis (H&E) revealed extensive epithelial damage, mucosal erosion, heavy inflammatory infiltration, and depletion of goblet cells in the UC group (Figure 5H). Free Lf, Lf/ASKP‐STMP, and Lf/ASKP‐Fe all alleviated colonic injury to varying degrees: the epithelial and crypt structures were partly restored, the mucosal integrity was improved, the goblet‐cell function was recovered and the inflammatory infiltration was decreased. The Lf/microgels formulations produced more pronounced effects than free Lf. The abundant brown MPO‐positive deposits in UC colons were markedly reduced by both microgel treatments, indicating an effective suppression of neutrophil infiltration.

To explore underlying molecular mechanisms, the TLR4/MyD88/NF‐κB inflammatory signaling axis was examined. qPCR showed significant upregulation of TLR4, MyD88, and NF‐κB mRNA in UC model colons (^*^ *p* < 0.05, Figure 5I). Lf/ASKP‐STMP treatment significantly downregulated all three transcripts (^*^ *p* < 0.05), suggesting that colon‐released Lf attenuates pathway activation and thus mitigates the inflammatory cascade. Tight junction (TJ) proteins were next evaluated because of their central role in barrier integrity [57]. UC was associated with marked reductions in ZO‐1, Claudin‐1, and Occludin expression, consistent with increased barrier permeability (Figure 5J). Colonic delivery of Lf by Lf/ASKP‐STMP significantly upregulated these TJ proteins, restored the tight‐junction barrier function, and recovered the mucosal barrier function compared with Lf/ASKP‐Fe, thereby limiting spread of the inflammation and alleviating UC symptoms. In summary, Lf/ASKP‐STMP provides sustained, colon‐localized release of Lf, and effective reduction of colonic dysfunction. This colonic sustained release resulted in a superior reduction in DAI and MPO, greater suppression of inflammatory cytokines, and more robust restoration of colon morphology and TJ protein expression in the UC model.

**FIGURE 6:**
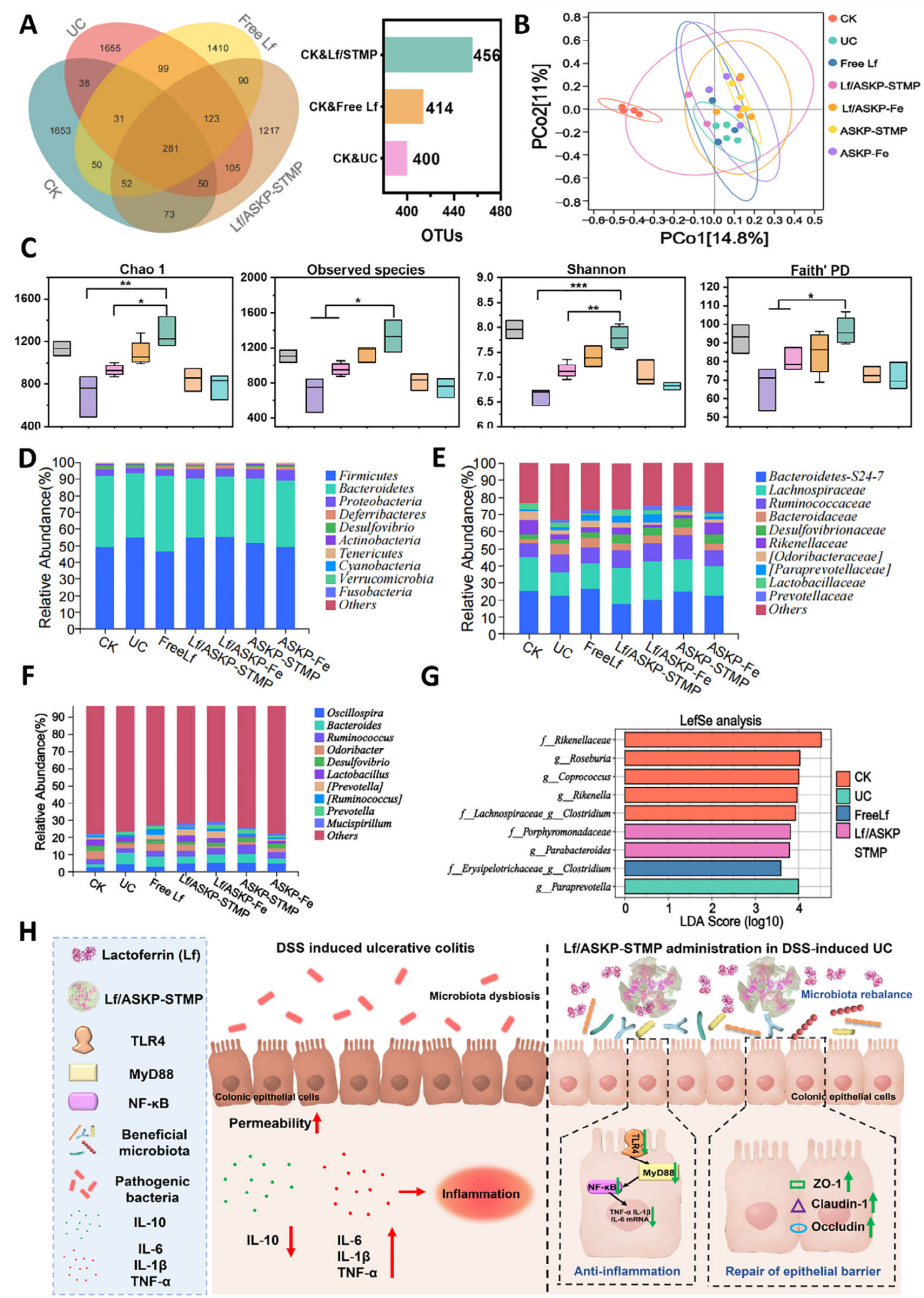
Microbiota remodeling by colon‐targeted Lf mediates therapeutic efficacy in ulcerative colitis. (A) Venn diagram of overlapping operational taxonomic units (OTUs) among groups. (B) Principal coordinates analysis (PCoA) of gut microbiota composition. (C) α‐diversity indices: Chao1, Observed species, Shannon, and Faith's PD, and (^*^ *p* < 0.05, ^**^ *p* < 0.01, ^***^ *p* < 0.001). (D‐F) Relative abundance of gut microbiota at the phylum, family, and genus level (top 10 taxa shown). (G) LEfSe analysis identifying differentially enriched taxa (LDA score >3.0). (H) Proposed mechanism: Colon‐targeted Lf delivery ameliorates colitis through microbiota modulation and suppression of the TLR4/NF‐κB pathway, restoring epithelial barrier integrity.

Recent studies showed that UC could lead to gut microbiota dysbiosis [58]. To evaluate the effect of Lf formulations on gut microbiota modulation, 16S rRNA sequencing was performed on cecal contents. The Venn diagram showed that Lf/ASKP‐STMP shared the highest number of OTUs, being more than free Lf or the UC group (Figure 6A). The β‐diversity analysis (Figure 6B) further revealed that Lf/ASKP‐STMP clustered most closely with CK, indicating a stronger restoration effect on overall community structure compared with Lf/ASKP‐Fe and free Lf. The α‐diversity indices were consistently higher in the treatment groups than UC group, and especially for Lf/ASKP‐STMP was approaching CK levels (Chao1, Observed species, Shannon, and Faith's PD; Figure 6C), highlighting its superior ability in restoring microbial richness and diversity. Examination of the microbiota at multiple taxonomic levels revealed treatment‐specific alterations in community composition. At the phylum level, *Firmicutes* and *Bacteroidetes* were substantially altered in the UC group (Figure 6D), while *Proteobacteria* were enriched, which is commonly associated with intestinal inflammation [59, 60]. At the family and genus level (Figure 6E,F), inflammation‐associated families such as *Desulfovibrionaceae* and *Rikenellaceae* were increased in the UC group, whereas beneficial commensal taxa such as *Lachnospiraceae* and *Ruminococcaceae* decreased [61, 62, 63]. Marked adjustments in microbial community structure were observed after Lf treatments. The Lf/ASKP‐STMP group showed a composition more similar to CK, characterized by restoration of beneficial bacterial families (*Lachnospiraceae*, *Ruminococcaceae*) and reductions of inflammation‐associated lineages. Lf/ASKP‐Fe and ASKP‐STMP without Lf also exerted modulatory effects on beneficial bacterial families, which may reflect differences in intrinsic prebiotic activity of ASKP natural polysaccharides. LEfSe analysis (LDA>3.0, Figure 6G) identified distinct microbial signatures across groups. The UC group was enriched in inflammation‐associated bacteria such as *Erysipelotrichaceae_Clostridium* and *Paraprevotella*, whereas Lf/ASKP‐STMP promoted colonization of *Porphyromonadaceae* and *Parabacteroides*, which are known for anti‐inflammatory properties in DSS‐induced colitis [64, 65, 66]. Importantly, because Lf/ASKP‐STMP releases Lf mainly in the colon, higher colonic Lf concentration likely inhibited the harmful bacteria (e.g., *Desulfovibrio*) and enriched the beneficial bacteria (e.g., *Parabacteroides* and *Oscillopsia*) [62, 67]. In contrast, free Lf showed weaker effects due to the early digestion and low colonic Lf availability. These results indicate that colonic targeted delivery of anti‐pathogenic Lf by ASKP‐STMP showed the most obvious colitis alleviation effect by improving intestinal epithelial barrier permeability and reshaping gut microbiota balance (Figure 6H).

## 3 Discussion

In this study, two ASKP‐based microgels for specific delivery of Lf to targeted sites such as the small intestine or colon were evaluated. The Lf of 78 kDa was protected by both microgels under simulated gastric conditions and responsively released Lf at either the small intestine when encapsulated by ASKP‐Fe microgels or at the colon by ASKP‐STMP microgels. Notably, these are food‐grade formulations and this work first time reports the micron‐sized ASKP microgels derived from seeds of the sand‐fixing plant *Artemisia sphaerocephala*. This enhanced gastric stability may be attributed to the compact gel network serving as a physical barrier reducing enzymatic (pepsin) accessibility. Besides, LfR‐mediated cellular uptake by epithelial cells was detected after Lf release from microgels, supporting the effective receptor‐dependent endocytosis. Moreover, Lf/ASKP‐Fe had an efficient Lf small intestinal absorption and high bioavailability of ≈6‐fold higher than free Lf resulting in more Lf entering into the blood circulation and accumulating in the liver with a high hepatic protective function against alcoholic liver injury. Lf/ASKP‐STMP delivered more Lf in the colon, causing an alleviation effect in an ulcerative colitis model. In addition, serum Immunoglobulin E (IgE) levels remained in the normal range during 16 days oral Lf formulation treatment, indicating no allergic risk. Both ASKP and Lf are food‐grade materials with authoritative national and international safety certifications, and the mild crosslinking preparation process ensures the oral safety of the delivery system. Collectively, these results show that food‐grade ASKP microgels protect Lf, preserve its active/receptor‐binding sites largely reach the intestinal tract, and markedly maximize in vivo health benefits; the platform is also applicable to improve other functional ingredients’ oral bioavailability and efficacy. ASKP microgels are thus promising as intestinal site‐specific delivery platform for protecting bioactive proteins on demand.

The rational design of a single natural polysaccharide ASKP through two distinct crosslinking strategies to achieve intestinal site‐specific protein release is a cost‐effective and clinically promising strategy. Fe^3^^+^ coordination enabled rapid, small intestine‐targeted release of Lf, thereby enhancing systemic absorption, hepatic accumulation, and bioavailability. Conversely, covalent STMP crosslinking facilitated enabled sustained colonic Lf delivery, which promoted microbiota modulation and ameliorated inflammatory responses. Crucially, this is the first demonstration that different cross‐linking chemistries applied to the same ASKP polysaccharides backbone enable site‐specific intestinal delivery, producing distinct gut‐region therapeutic outcomes (gut‐liver axis modulation vs. colonic anti‐inflammation and microbiota reshaping). Microgel protection preserved Lf structural integrity and its receptor‐binding domain, thereby restoring LfR/clathrin‐mediated endocytosis—a process essential for physiological Lf activity. The pharmacokinetic, tissue distribution, and proteomic analyses further revealed distinct Lf metabolic fates and host proteome alterations, linking site‐specific delivery to organ‐specific modulation of lipid metabolism, antioxidant pathways, and inflammatory signaling.

Altogether, the ASKP microgel platform holds promise as a generalizable strategy for intestinal‐segmental oral delivery of bioactive proteins. This study only conducts a preliminary mechanistic exploration by verifying core pathways at the gene levels without screening key downstream targets, and in‐depth omics and cellular experiments will be performed in future research to clarify the precise molecular regulatory mechanism. Future studies can apply these microgel platforms to load other bioactive peptides/proteins such as GLP‐1 or insulin and to study their therapeutic efficacy in vivo. Moreover, detailed mapping of proteomic and metabolic patterns at the tissue‐level pharmacokinetic scale, in conjunction with the gut‐liver axis, will clarify the mechanism of organ‐specific efficacy and accelerate their clinical applications.

## 4 Experimental Section

### 4.1 Materials

The following reagents were used in this study: *Artemisia sphaerocephala* gum (Shanghai Yuanye Bio‐Technology Co., Ltd.), ferric chloride (Shanghai Aladdin Biochemical Technology Co., Ltd.), sodium trimetaphosphate (Shanghai Macklin Biochemical Technology Co., Ltd.), liquid paraffin (Shanghai Hushi Co., Ltd.), Span 80, pepsin, trypsin, Tween 80, and CCK‐8 kit (Sigma‐Aldrich Co., LLC.), methanol and concentrated hydrochloric acid (Beijing Chemical Works), n‐hexane (Shanghai Macklin Biochemical Technology Co., Ltd.), absolute ethanol and potassium dihydrogen phosphate (Shanghai Hushi Co., Ltd.), sodium hydroxide (Beijing Chemical Works), chromatographic grade acetonitrile (Shanghai Aladdin Biochemical Technology Co., Ltd.), bLf ELISA kit (Beijing Borui Changyuan Technology Co., Ltd), anti‐fluorescence quenching mounting medium (with DAPI) and dextran sulfate sodium (DSS) (Beijing Solarbio Science & Technology Co., Ltd.), Cy5 dye (Beijing Fanbo Biochemical Co., Ltd.), DAPI and AF488‐conjugated wheat germ agglutinin (WGA) dyes (Thermo Fisher Scientific Inc.), human colon adenocarcinoma cells (Caco‐2) were purchased from Peking Union Medical College Cell Resource Center (received in 2023.11, RRID:CVCL_0025, the cell line was contamination‐free by STR test), fetal bovine serum (FBS), MEM medium, 0.25% trypsin‐EDTA, and Opti‐MEM reduced‐serum medium (Gibco, Thermo Fisher Scientific Inc.), phalloidin‐FITC (Thermo Fisher Scientific Inc.), Lipofectamine RNAiMAX transfection reagent (Thermo Fisher Scientific Inc.), universal RNA extraction kit, Evo M‐MLV RT Premix kit, and SYBR Green Pro Taq HS qPCR kit (Hunan Accura Biology Engineering Co., Ltd.), PCR primers (Beijing Liuhe BGI Technology Co., Ltd.), mouse MPO and cytokine (IL‐6, IL‐1β, TNF‐α, IL‐10) ELISA kits (Beijing Biorab Technology Co., Ltd.), H&E staining kit (Wuhan Servicebio Technology Co., Ltd.), RNeasy kit (Qiagen GmbH), mouse liquid feed (Jiangsu Xietong Medical Bioengineering Co., Ltd.), food‐grade alcohol (Gengma Hualin Alcohol Co., Ltd.), assay kits for AST, ALT, total SOD, GSH/GSSG, total cholesterol, HDL‐C, LDL‐C, and triglycerides (Nanjing Jiancheng Bioengineering Institute).

### 4.2 Characterization of ASKP Properties

The monosaccharide composition of ASKP was determined by high‐performance liquid chromatography (HPLC) following established protocols for acid hydrolysis and derivatization. The ζ‐potential of aqueous ASKP solutions in deionized water was measured at 25°C using a Malvern Zetasizer Nano ZS instrument (Malvern Panalytical Ltd., UK).

### 4.3 Characterization of ASKP Microgels

ASKP microgels were fabricated via an emulsion‐templating approach, employing two distinct crosslinking strategies: covalent crosslinking with sodium trimetaphosphate (STMP) and ionic crosslinking with Fe^3^^+^ ions. The preparation involved emulsifying a pre‐sonicated 4% ASKP solution in a paraffin/Span 80 oil phase, followed by crosslinking under alkaline conditions for STMP or at ambient temperature for FeCl_3_. The resulting microgels were collected by centrifugation, washed with hexane and methanol, and lyophilized. Morphological evaluation was performed using optical microscopy and scanning electron microscopy (SEM). Elemental composition (C, O, P, Fe) was analyzed by energy‐dispersive X‐ray spectroscopy (EDS). Mean particle size was quantified from micrographs using ImageJ software, and surface charge was determined via ζ‐potential measurements with a Malvern Zetasizer. Chemical structures of crosslinked microgels were further characterized by Fourier transform infrared (FTIR) spectroscopy with KBr‐pelletized samples.

### 4.4 Preparation and Characterization of Lf‐Loaded ASKP Microgels

Lf was loaded into ASKP microgels via two methods: electrostatic adsorption into pre‐formed STMP‐crosslinked microgels (Lf/ASKP‐STMP), and direct encapsulation during Fe^3^^+^ crosslinking (Lf/ASKP‐Fe). The encapsulation was performed at 4°C with shaking, followed by collection through centrifugation and lyophilization. Encapsulation efficiency (EE) and loading efficiency (LE) were determined using an Lf ELISA kit. For Lf/ASKP‐STMP, unbound Lf in the supernatant was measured. For Lf/ASKP‐Fe, Lf was released by disintegration in potassium dihydrogen phosphate buffer (0.05 M, pH 6.8) and quantified. EE and LE were calculated accordingly. Surface hydrophobicity was evaluated by fluorescence spectroscopy with an ANS probe, recording emission spectra (400‐600 nm) at an excitation wavelength of 380 nm. The surface hydrophobicity index (H_0_) was derived from the slope of the fluorescence intensity vs. protein concentration.

### 4.5 Release Properties of Lf‐Loaded ASKP Microgels

The structural stability of encapsulated Lf was evaluated by SDS‐PAGE. Released Lf was extracted from lyophilized microgels by grinding and centrifugation, then electrophoresed under non‐reducing conditions and visualized by Coomassie Blue staining. In vitro release profiles were assessed in simulated gastric (SGF, pH 1.2, with pepsin) and intestinal (SIF, pH 6.8, with pancreatin) fluids. Microgel integrity and Lf release were monitored using UV spectrophotometry, ELISA, and fluorescence microscopy with Cy5‐labeled Lf. Supernatants and digested microgels were sampled at intervals and analyzed for Lf content and structural consistency.

### 4.6 Cellular Uptake Study of Lf‐Loaded ASKP Microgels

Caco‐2 cells (passages 20–55) were cultured in MEM containing 10% FBS and antibiotics at 37°C under 5% CO_2_. Four types of Lf samples with equivalent content were prepared and labeled with Cy5: Untreated Lf: untreated native Lf solution; Free Lf: unencapsulated Lf digested in SGF (pH 1.2, 2 h), with digestion terminated by pH adjustment to 6.0; ASKP‐STMP‐Lf: Lf released from STMP‐crosslinked microgels after SGF digestion; ASKP‐Fe‐Lf: Lf released from Fe^3^^+^‐crosslinked microgels after SGF digestion. Unbound dye was removed via dialysis. For qualitative uptake analysis, cells were treated with Cy5‐labeled samples, fixed, and stained with FITC‐phalloidin and DAPI, then imaged by confocal microscopy. For quantitative analysis, cells were trypsinized after treatment and analyzed by flow cytometry to measure Cy5 fluorescence. Roles of lactoferrin receptor (LfR) and clathrin were investigated using siRNA knockdown. Caco‐2 cells were transfected with siRNA targeting LfR or clathrin heavy chain (CLTC) (siRNA sequences are listed in Table S5) using Lipofectamine RNAiMAX. After 48 h, knockdown efficiency was validated by RT‐qPCR using primer sequences shown in Table S6. Cellular uptake of Cy5‐Lf samples in transfected cells was assessed using flow cytometry.

### 4.7 Pharmacokinetic Study

Male Sprague‐Dawley rats were acclimatized under controlled conditions. All animal procedures were approved by the Beijing Laboratory Animal Ethics Committee (Approval No. AW92804202‐4‐3). Rats were fasted for 24 h prior to the experiment and then randomly divided into three groups (*n* = 5) for oral administration: Free Lf, Lf/ASKP‐Fe, and Lf/ASKP‐STMP. The dose for each rat was calculated based on Lf content at 80 mg/kg body weight, administered in a volume of 1 mL. After administration, fasting continued for 8 h. Blood samples (100 µL) were collected via cardiac puncture at designated time points and placed in anticoagulant tubes. The serum was separated by centrifugation (3000 rpm, 15 min, 4°C) and stored at −80°C. Lf concentrations in serum were determined by a bovine Lf‐specific ELISA kit. Due to cross‐reactivity of the ELISA kit with endogenous rat Lf, a baseline correction was applied to accurately assess the pharmacokinetics of exogenous bovine Lf. For each rat, the pre‐dose (0 h) serum Lf concentration (*C_0_*) was considered as its individual endogenous level and was subtracted from the total concentration measured at each post‐dose time point (*C_total(t)_*) to obtain the net increase in bovine Lf concentration (*C_t_ = C_total(t)_—C_0_*). Any resulting negative Ct values were treated as zero in subsequent analyses. Pharmacokinetic parameters (*C_max_, T_max_, AUC_0_‐_t_*) were derived using GraphPad Prism. Relative bioavailability (*F_rel_*, %) was calculated according to the following formula:

(1) $$F_{rel}\;\left(\%\right)=\frac{AUC\left(test\right)\times Dose\left(test\right)}{AUC\left(ref\right)\times Dose\left(ref\right)}\;$$

where *AUC (test)* and *Dose (test)* refer to the AUC and dose for the Lf/ASKP‐Fe or Lf/ASKP‐STMP groups, and *AUC (ref)* and *Dose (ref)* refer to the AUC and dose for the Free Lf group.

### 4.8 Proteomic Analysis of Serum and Liver Tissues

Free Lf and Lf/ASKP‐Fe were orally administered to rats and mice by gavage. Serum samples from rats and liver tissues from mice were collected at *T_max_* based on previous pharmacokinetic studies. Subsequently, rat serum and mouse liver samples were subjected to protein digestion and peptide purification, followed by high‐resolution proteomic analysis using the Astral mass spectrometer with data‐independent acquisition (DIA).

### 4.9 In Vivo Release and Biodistribution

Male C57BL/6J mice were used under the ethical approval (Approval No. AW92804202‐4‐3). For tissue distribution, mice received oral gavage of Cy5‐labeled Free Lf, Lf/ASKP‐STMP, or Lf/ASKP‐Fe (100 mg Cy5‐Lf/kg). Tissues (intestine, heart, liver, spleen, lung, kidney, brain) and colonic content were collected at scheduled times. Paraffin sections of intestinal segments were stained with DAPI and AF488‐WGA and imaged by fluorescence microscopy. Lf in homogenates was quantified by ELISA. For whole‐body biodistribution, organs and the GI tract were excised at various time points and imaged using an in vivo imaging system (Ex/Em: 649/670 nm).

### 4.10 Effects of Lf‐Loaded Microgels on Alcohol‐Induced Liver Injury in Mice

Forty‐nine male C57BL/6 mice (8 weeks old) were randomly assigned to seven groups (*n* = 7): Model, Free Lf, Lf/ASKP‐STMP, Lf/ASKP‐Fe, ASKP‐STMP, ASKP‐Fe, and Control (CK). All animal experiments were approved by the China Agricultural University Laboratory Animal Welfare and Ethics Committee (Approval No. AW92804202‐4‐4). Mice in Groups 1–6 were fed a Lieber‐DeCarli ethanol liquid diet, gradually acclimatized to 5% ethanol over several days. The CK group received an isocaloric control diet. Throughout the experiment, groups received daily oral gavage (200 µL) of normal saline (Model and CK), Free Lf (300 mg/kg bw), Lf/ASKP‐STMP (300 mg/kg bw), Lf/ASKP‐Fe (300 mg/kg bw), ASKP‐STMP, or ASKP‐Fe [22]. Body weight was recorded daily. On day 16, an acute ethanol challenge (5 g/kg bw) was administered to Groups 1–6; the CK group received maltodextrin. Mice were euthanized 9 h later. Blood and liver tissues were collected for further analysis. Serum levels of AST, ALT, T‐CHO, TG, LDL‐C, HDL‐C and IgE were measured using commercial kits. Inflammatory cytokines (IL‐6, IL‐1β, TNF‐α, IL‐10) were quantified via ELISA. Hepatic SOD activity and glutathione levels (T‐GSH and GSSG) were determined using commercial assay kits. mRNA expression of alcohol metabolism‐related genes was analyzed by qPCR using primers listed in Table S7.

### 4.11 Effects of Lf‐Loaded Microgels on Ulcerative Colitis in Mice

Forty‐nine male BALB/c mice (8 weeks old) were randomly divided into seven groups (*n* = 7): Model, Free Lf, Lf/ASKP‐STMP, Lf/ASKP‐Fe, ASKP‐STMP, ASKP‐Fe, and Control (CK). Colitis was induced by administering 3.5% dextran sulfate sodium (DSS) in drinking water for 7 days, except for the CK group which received normal water. After 7 days of DSS water induction, normal water was replaced with 200 µL of physiological saline (UC group), Free Lf, Lf/ASKP‐Fe, Lf/ASKP‐STMP, ASKP‐Fe, and ASKP‐STMP by gavage in each group, once a day for five days. The relative concentration of Lf in each group was 100 mg/kg, and the CK group mice were given normal saline water by gavage as a control [68]. All procedures were approved by the China Agricultural University Laboratory Animal Welfare and Ethics Committee (Approval No. AW90504202‐4‐2). The Disease Activity Index (DAI) was scored daily based on body weight loss, stool consistency, and fecal bleeding according to Table S8. Mice were euthanized six hours after the final gavage. Blood, colon, spleen, and cecal contents were collected. Colon length and spleen weight were measured. Colon segments were either fixed for histology or snap‐frozen for molecular analysis. Serum levels of myeloperoxidase (MPO) and cytokines (IL‐6, IL‐1β, TNF‐α, IL‐10) were quantified using commercial ELISA kits. Colon sections were stained with H&E for pathological evaluation. Immunohistochemistry (IHC) was performed to assess specific protein localization and expression. Total RNA was extracted from colon tissue. The mRNA expression of target genes was analyzed by qPCR using primers listed in Table S9, with GAPDH as the reference gene.

### 4.12 Gut Microbiota Analysis

Cecal contents were analyzed by 16S rRNA sequencing. Alpha diversity (Chao1, Shannon, Faith's PD, Observed species) and beta diversity (PCoA) were assessed. Taxonomic composition at phylum and genus levels was determined.

### 4.13 Statistical Analysis

Statistical analysis was conducted using SPSS (IBM Corp.). Data are presented as mean ± standard deviation, unless indicated. Significance levels are denoted as ^*^ *p* < 0.05, ^**^ *p* < 0.01, and ^***^ *p*<0.001.

## Author Contributions

**Huiling Yan** and **Yixuan Li** contributed equally to this work. **Huiling Yan** and **Yixuan Li** performed the experiments, curated data, performed formal analysis and visualization, worked with the software, developed methodology and wrote the original draft. **Shanan Chen**, **Pengcheng Du**, **Kaiwen Wu** and **Hui Zhang** performed the experiments, conducted validation, and reviewed and edited the manuscript. **Kasper Hettinga**, **Lina Zhang**, **Gergely Toldi** and **Fazheng Ren** conducted validation and reviewed and edited the manuscript. **Yuan Li** developed conceptualization, developed methodology, reviewed and edited the manuscript, supervised the research, provided resources, and acquired funding.

## Funding

This work was financially supported by National Key Research and Development Program of China (2024YFF1106500, 2024YFF1106504) and the Pin‐duo‐duo China Agricultural University Research Fund (PC2023B01007).

## Conflicts of Interest

The authors declare no conflicts of interest.

## Supporting information




**Supporting File**: advs75697‐sup‐0001‐SuppMat.docx.

## Data Availability

The data that support the findings of this study are available from the corresponding author upon reasonable request.
