# Supplementary material for: A Microgel Platform Enables Site‐Specific Intestinal Delivery of Lactoferrin, Improving its Bioavailability for Targeted Alleviating Liver Injury and Colitis
Source: Adv Sci (Weinh). 2026 May 13;13(45):e75697. doi: 10.1002/advs.75697 (PMC13335855; doi:10.1002/advs.75697)
Supplement: Supplementary file 1 [file ADVS-13-e75697-s001.docx]

Supporting Information

**A microgel platform enables site-specific intestinal delivery of lactoferrin, improving its bioavailability for targeted alleviating liver injury and colitis**

*Huiling Yan**^1,2,#^,* *Yixuan Li**^1,#^,* *Shanan Chen^1,2^, Pengcheng Du^1,2^, Kaiwen Wu^1,2^, Hui Zhang^1,2^, Kasper* *Hettinga^3^, Lina Zhang^4^, Gergely Toldi^5^, Fazheng Ren^1^, Yuan Li^1,2,^**

\# Huiling Yan and Yixuan Li contributed equally to this work.

*Corresponding author Prof. Yuan Li, E-mail: yuanli@cau.edu.cn

Figure S1 Chromatogram of the monosaccharide fraction of ASKP (1 mannose, 2 ribose, 3 rhamnose, 4 glucuronic acid, 5 galacturonic acid, 6 glucose, 7 galactose, 8 xylose, 9 arabinose, 10 fucose)

Table S1 Table of monosaccharide fractions of ASKP.

| Peak No. | Retention time | Compound name | Peak area | Peak height | Peak area (%) |
| --- | --- | --- | --- | --- | --- |
| 1 | 13.669 | mannose | 10651457 | 572691 | 46.776 |
| 2 | 17.792 | ribose | 14894 | 677 | 0.065 |
| 3 | 18.749 | rhamnose | 123182 | 4019 | 0.541 |
| 4 | 22.339 | glucuronic acid | 1269601 | 43657 | 5.575 |
| 5 | 24.559 | galacturonic acid | 70117 | 1511 | 0.308 |
| 6 | 29.331 | glucose | 6492467 | 168806 | 28.512 |
| 7 | 33.636 | galactose | 3798567 | 86583 | 16.681 |
| 8 | 35.403 | xylose | 33892 | 792 | 0.149 |
| 9 | 36.752 | arabinose | 282342 | 5503 | 1.240 |
| 10 | 40.018 | fucose | 34622 | 548 | 0.152 |
| Total | / | / | 22771141 | 884787 | 100.000 |

Table S2 The ζ-potentials of ASKP, ASKP-Fe and ASKP- STMP.

| Sample | ζ-potential（mV） |
| --- | --- |
| ASKP | -31.12 ±1.04 |
| ASKP-Fe | -29.72 ±1.53 |
| ASKP-STMP | -45.98 ± 1.31 |

Figure S2 Fluorescence microscopy of Lf(Cy5)-loaded ASKP-Fe and ASKP-STMP microgels.

Figure S3 Encapsulation efficiency (EE) and loading efficiency (LE) of Lf in ASKP-Fe microgels (A) and ASKP-STMP microgels (B) at varying ASKP: Lf ratios.

Figure S4 The ζ-potentials of Lf in ASKP-Fe microgels (A) and ASKP-STMP microgels (B) at varying ASKP: Lf ratios.

Figure S5 SDS-PAGE plots of two microgel breaking spheres releasing Lf and standard Lf (1. marker; 2. Lf standard; 3. Lf of Lf/ASKP-STMP; 4. Lf of Lf/ASKP-Fe)

Figure S6 Fluorescence spectra and surface hydrophobicity.

Figure S7 Fluorescence images of Lf/ASKP-Fe during simulated gastrointestinal digestion.

Figure S8 Fluorescence images of Lf/ASKP-STMP during simulated gastrointestinal digestion.

Figure S9 Fluorescent labelling of Lf after SGF digestion.

Figure S10 CLSM images of endocytosed by Caco-2 cells in each group of Lf samples.

Figure S11 LfR and clathrin mRNA expression in LfR and clathrin siRNA transfected Caco-2 cells.

Figure S12 Intestinal sections of Lf distribution in the gastrointestinal tract (red: Cy5-labelled Lf; green: FITC-WGA-labelled mucus; blue: DAPI-labelled cell nucleus).

Table S3 Values of Lf pharmacokinetic parameters in different groups.

| pharmacokinetic parameters | Lf | Lf/ASKP-Fe | Lf/ASKP-STMP |
| --- | --- | --- | --- |
| *T_max_* (h) | 1.50^c^ | 2.00^a^ | 1.75^b^ |
| *C_max_* (ng/mL) | 41.4^c^ | 146.3^a^ | 78.8^b^ |
| *AUC_0-8_* (ng h/mL) | 46.6^c^ | 276.9^a^ | 158.3^b^ |
| *F_rel_* (%) | 100%^c^ | 593%^a^ | 339%^b^ |

Values with different superscript letters denote significant differences at *p*<0.01.

Figure S13 Quantification of intact Lf in organs and colonic content by ELISA.

Figure S14 Quantification of serum IgE after 16-day oral Lf administration by ELISA.

Table S4 Proteomic identification results of serum

| Protein ID | Protein  Description | Sequence  Length | Molecular  Weight.KDa. | Relative Abundance (a.u.) | | |
| --- | --- | --- | --- | --- | --- | --- |
|  |  |  |  | Blank | Free Lf | Lf/ASKP-Fe |
| P24627 | Lactotransferrin | 708 | 78.056 | 15437900 | 42897425 | 81548500 |
| Q8MII0 | Lactotransferrin (Fragment) | 355 | 38.861 | 17638267 | 51503425 | 96935775 |

Figure S15 Relative abundance of Lf and its fragment across groups in serum.

Figure S16 Fold change of Lf and its fragment across groups in serum.

Figure S17 Relative abundance of Lf and its fragment across groups in liver.

Figure S18 Fold change of Lf across groups in liver.

Figure S19 Volcano plot (Free Lf vs Lf/ASKP-Fe) highlighting the top 10 up- and down-regulated differential proteins in serum.

Figure S20 Body weight changes during modeling (***p*<0.01, ****p*<0.001).

Figure S21 Liver coefficient of each group.

Figure S22 Serum ALT and AST levels (**p*<0.05, ***p*<0.01, ****p*<0.001).

Figure S23 Histopathological analysis: H&E staining and Oil Red O staining.

Figure S24 Serum levels of low-density lipoproteins (LDL) and high-density lipoproteins (HDL). (**p*<0.05, ***p*<0.01, ****p*<0.001)

Figure S25 Measurement of TNF-α and IL-10, indicators of inflammation in alcoholic liver injury. (**p*<0.05, ***p*<0.01)

Figure S26 Total glutathione (T-GSH) content in the liver. (**p*<0.05, ***p*<0.01, ****p*<0.001).

Figure S27 Schematic diagram of alcohol metabolism mechanism.

Figure S28 Schematic diagram of Nrf2 and PI3K Akt antioxidant pathways.

Figure S29 Symptoms and signs of mice in each group.

Figure S30 hotographs of faecal morphology of mice in each group

Table S5 siRNA sequence.

| **gene** | **sense strand（5’-3’）** | **antisense strand（5’-3’）** |
| --- | --- | --- |
| LfR siRNA1 | CGAAUGUCCUAGUGCAUUUTT | AAAUGCACUAGGACAUUCGTT |
| LfR siRNA2 | CGGUGAUCCCUGUGGUCUAUGTT | CAUAGACCACAGGGAUCACCGTT |
| LfR siRNA3 | GGAGUACAGAUGAGGCUAAUATT | UAUUAGCCUCAUCUGUACUCCTT |
| clathrin siRNA1 | GCUAGCAAAGUAAUUGCACTT | GUGCAAUUACUUUGCUAGCTT |
| clathrin siRNA2 | GCCCAAAUGUUAGUUCAAGAUTT | AUCUUGAACUAACAUUUGGGCTT |
| clathrin siRNA3 | CCUGUGUAGAUGGGAAAGAAUTT | AUUCUUUCCCAUCUACACAGGTT |

Table S6 qPCR primer sequences.

| **gene** | **sense strand（5’-3’）** | **antisense strand（5’-3’）** |
| --- | --- | --- |
| LfR | GGAGCGTTTTTGGAGAAAGCTGCA | ATTAACATTCTAGCTACTGGGT |
| clathrin | ATTCTGCCAATTCGTTTTCAGGA | GCTTTCAGTGCAATTACTTTGCT |
| GAPDH | GACTCATGACCACAGTCCATGC | AGAGGCAGGGATGATGTTCTG |

Table S7 Primers used in polymerase chain reaction.

| **target gene** | **Primer sequence** |
| --- | --- |
| CYP2E1 | F: 5ʹAGGCTGTCAAGGAGGTGCTACT 3ʹ |
|  | R: 5ʹ AAAACCTCCGCACGTCCTTCCA 3ʹ |
| ALDH | F: 5ʹ GCTGTTGTACCGATTGGCGGAT 3ʹ |
|  | R: 5ʹ GCGGAGACATTTCAGGACCATG 3ʹ |
| ADH | F: 5ʹ GCTATGGCTCTGCCGTCAAAGT 3ʹ |
|  | R: 5ʹ TGTCCACAGCAATGATCCTGGC 3ʹ |
| CAT | F: 5ʹ CGGCACATGAATGGCTATGGATC 3ʹ |
|  | R: 5ʹ AAGCCTTCCTGCCTCTCCAACA 3ʹ |
| Nrf2 | F: 5ʹ CAGCATAGAGCAGGACATGGAG 3ʹ |
|  | R: 5ʹ GAACAGCGGTAGTATCAGCCAG 3ʹ |
| keap1 | F: 5ʹ CATCCACCCTAAGGTCATGGA 3ʹ |
|  | R: 5ʹ GACAGGTTGAAGAACTCCTCC 3ʹ |
| PI3K | F: 5ʹ CAAACCACCCAAGCCCACTACT 3ʹ |
|  | R: 5ʹ CCATCAGCAGTGTCTCGGAGTT 3ʹ |
| CPT1A | F: 5ʹ GGCATAAACGCAGAGCATTCCTG 3ʹ |
|  | R: 5ʹ CAGTGTCCATCCTCTGAGTAGC 3ʹ |
| GAPDH | F: 5ʹ GACTCATGACCACAGTCCATGC 3ʹ |
|  | R: 5ʹ AGAGGCAGGGATGATGTTCTG 3ʹ |

Table S8 Disease activity index assessment standards.

| **Score** | **Weight loss（%）** | **Fecal property** | **Hidden blood** |
| --- | --- | --- | --- |
| 0 | 0 | normal | normal |
| 1 | 0-5 | Soft but shaped | Weakly positive |
| 2 | 5-10 | soft stool | positive |
| 3 | 10-15 | diarrhea | Strong Positive |
| 4 | ＞15 | watery diarrhoea | Visible bloody stool to the naked eye |

Table S9 Primers used in polymerase chain reaction.

| **target gene** | **Primer sequence** |
| --- | --- |
| TLR4 | F: 5ʹ GATCTGAGCTTCAACCCCCTG 3ʹ |
|  | R: 5ʹ TCCACAGCCACCAGATTCTC 3ʹ |
| *NF-κB* | F: 5ʹ TGTCAACAGGGTAACCTACCA 3ʹ |
|  | R: 5ʹ ACATTCTTTTTGCCACTTTCC 3ʹ |
| MyD88 | F: 5ʹ ACCTGTGTCTGGTCCATTGCCA 3ʹ |
|  | R: 5ʹ GCTGAGTGCAAACTTGGTCTGG 3ʹ |
| *ZO-1* | F: 5ʹ GTTGGTACGGTGCCCTGAAAGA 3ʹ |
|  | R: 5ʹ GCTGACAGGTAGGACAGACGAT 3ʹ |
| *Claudin-1* | F: 5ʹ TGCCCCAGTGGAAGATTTACT 3ʹ |
|  | R: 5ʹ CTTTGCGAAACGCAGGACAT 3ʹ |
| *Occludin* | F: 5ʹ AAGTCAACACCTCTGGTGCC 3ʹ |
|  | R: 5ʹ TCATAGTGGTCAGGGTCCGT 3ʹ |
| GAPDH | F: 5ʹ GACTCATGACCACAGTCCATGC 3ʹ |
|  | R: 5ʹ AGAGGCAGGGATGATGTTCTG 3ʹ |
